# Lymph Node Flow Cytometry as a Prompt Recognition of Ultra Early Onset PTLD: A Successful Case of Rituximab Treatment

**DOI:** 10.1155/2015/430623

**Published:** 2015-03-24

**Authors:** Xiaofan Li, Nainong Li, Ting Yang, Zhizhe Chen, Jianda Hu

**Affiliations:** Department of Hematology, Fujian Institute of Hematology, Fujian Provincial Key Laboratory on Hematology, Fujian Medical University Union Hospital, Fuzhou 350001, China

## Abstract

Ultra early posttransplantation lymphoproliferative disorder (PTLD) is a rare and fatal complication after hematopoietic stem cell transplantation (HSCT). Here we report, by lymph node (LN) flowcytometry, that we early recognized ultra early PTLD after an HLA-matched sibling allo-HSCT followed by a successful treatment with anti-CD20 antibody (rituximab) in a patient in progress disease for angioimmunoblastic T-cell lymphoma (AITL). The patient was conditioned with a reduced intensity conditioning (RIC) regimen. One week after transplantation, the patient developed high fever, generalized fatigue, high Epstein-Barr virus (EBV) load, and LN enlargement. An LN lymphocyte suspension and peripheral blood flowcytometry was performed to find majority of LN lymphocytes highly expressed CD20. By highly suspicious PTLD, 4 doses of rituximab (375 mg/m^2^ qw) were given immediately followed by reducing and withdrawing immunosuppressant reagent. PTLD was later confirmed by pathology. The patient had good response to rituximab, showing absence of fever, reduction in LN size, and no detectable EBV-DNA. Twenty months after HSCT, the patient remains well without evidence of AITL and PTLD. The current report is one of the earliest cases of PTLD after HSCT. Taken together, by LN flowcytometry as a prompt recognition, rituximab can be an effective preemptive therapy for ultra early developed PTLD.

## 1. Introduction

Hematopoietic stem cell transplant (HSCT) provides a curative treatment to hematologic malignancies [1]. Posttransplant lymphoproliferative disorder (PTLD) is a fatal complication in spite of its low incident rate. PTLD incidence ranges from 1 to 20%, depending on disease, age, Epstein-Barr virus (EBV), immunosuppression, and the transplanted allograft [2].

In solid organ transplantation, the incident rate is higher, which usually happens one year after transplantation. Accordingly, early onset of PTLD refers to those that happen within the first year after transplant. Khedmat and Taheri defined those that happen within the first month after transplant “ultra early onset PTLD” [3].

In bone marrow transplantation, PTLD usually happens 70–90 days after HSCT and is often related to EBV [4]. Given the time interval between transplantation and onset of PTLD in solid organ transplantation, it is reasonable to define those that happen within “100 days” after HSCT “early onset” PTLD. Similarly, “ultra early onset” of PTLD refers to those that happen within 10 days after HSCT.

PTLDs represent a heterogeneous group of lymphoproliferative diseases, which show a spectrum of clinical, morphological, and molecular genetic features ranging from reactive polyclonal lesions to frank lymphomas. Most cases of PTLD include a spectrum of B-cell hyperproliferative states, among which diffuse large B cell lymphoma can be one of the pathology manifestation [5]. Also, HLA-mismatching, T-cell depletion, and the use of ATG are the risk factors of PTLD [6]. In clinic, PTLD could present in a myriad of ways. Those recipients showing adenopathy, mass lesions, fever, and unexplained pain after HSCT are required for diligent surveillance to diagnose PTLD [7]. Withdrawing/reducing immunosuppressant reagent, monoclonal anti-B-cell antibodies (such as rituximab) and interferon-*α* (IFN-*α*) are the treatment of this disease, as well as donor lymphocyte infusion (DLI) (such as EBV-specific T cells) [8–10].

In general, PTLD may be difficult to distinguish from organ rejection and infection. The successful treatment relies on early recognition. Tissue biopsy is the gold standard for PTLD [11]. However, pathology results take time to deliver. Herein, we report, by lymph node flow cytometry, that we promptly recognised and successfully treated an ultra early onset of PTLD after an HLA-matched sibling allo-HSCT (10/10) followed by a successful treatment with anti-CD20 antibody (rituximab) in a patient in progress disease for angioimmunoblastic T-cell Lymphoma (AITL).

## 2. Case Report

**Figure 1:**
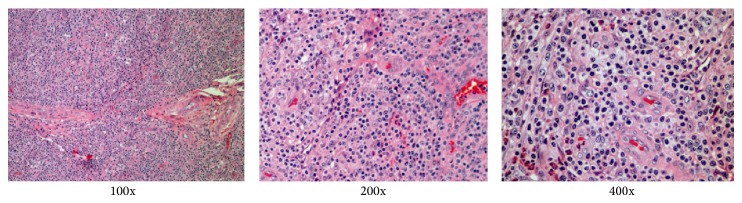
Pathology of AITL. The pathology slides were stain of H&E by standard procedure. Pathology of AITL was shown separately (100x, 200x, and 400x).

A 54-year-old Chinese Han male with relapse of angioimmunoblastic T-cell Lymphoma (AITL) was admitted to Fujian Medical University Union Hospital to perform allo-HSCT (Figure 1). He had a transient remission from AITL after 4 courses of chemotherapy (2 course of CHOP, 2 course of DHAP) followed by auto-HSCT (conditioning regimen: BEAM; 3.3 × 10^6^ cells/kg of CD34-positive cells). Six months later, the patient showed progression. He suffered from lymphadenopathy and B symptoms. With two courses of GDP regimen chemotherapy, the size of lymph node was reduced. However, a BM biopsy analysis was performed to show a BM involvement. Accordingly, a sibling PBSCT was performed.

In view of the patients performed status and short-duration of high-dose chemotherapy, a reduced-intensity conditioning of fludarabine (30 mg/m^2^ for three days), busulfan (0.8 mg/kg for three days), and rabbit antithymocyte globulin (ATG; 10 mg/kg) was applied to precondition the patient. G-CSF-mobilized (300 *μ*g twice a day for 5 days) peripheral blood (PB) stem cells containing 6.8 × 10^6^/kg of CD34-positive cells were transplanted from an HLA matched (10/10) female sibling. After HSCT, MMF and CsA were used to prevent GVHD.

**Figure 2:**
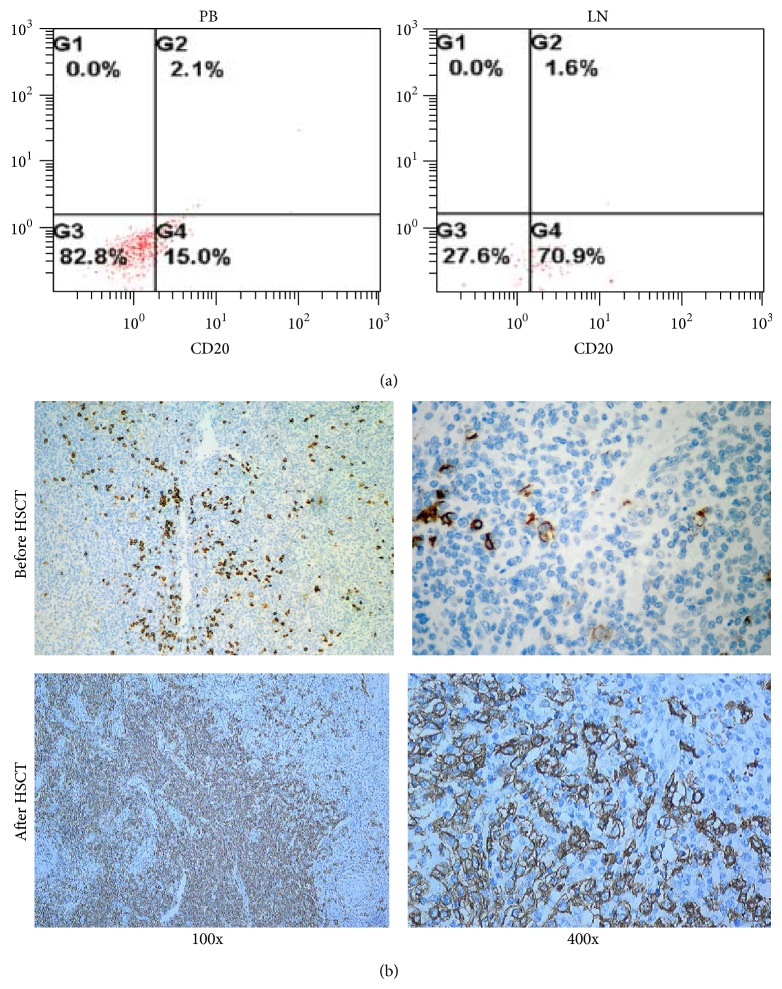
CD20 expression in the LN. The patient underwent lymph node (LN) biopsy. (a) After the operation, LN was grinded to prepare LN suspension. The lymphocytes of LN and peripheral blood (PB) were stained for CD2, CD3, CD5, CD7, HLA-DR, CD10, CD34, CD8, CD23, FMC7, CD79b, CD79a, CyCD3, and CD20. A representative flow pattern of CD20 was shown. (b) Further confirmation of CD20 expression in pathology slides. The pathology slides were stain of anti-CD20 by standard immunohistochemistry staining. Pathology was shown before and after HSCT separately (100x and 400x).

On day 7, the patient showed a recurring high fever above 38.5°C, was extremely fatigued, and had lymph node enlargement. Three days of antibiotic treatment did not show any effect and EBV-DNA showed high level copies. A CT scan was performed to evaluate the extent of the disease. The lymph node enlargement involved subclavian, axillary, popliteal fossa, and inguinal superficial lymph nodes as well as the mediastinal and abdominal lymph nodes. Thus, a lymph node biopsy was performed immediately. Although tissue biopsy is the gold standard for diagnosing PTLD, pathology results take time to deliver. We hypotheses that Lymph node flow cytometry could be a prompt recognition method of PTLD. Thereafter, by grinding the lymph node (LN), we compare the lymphocytes between the LN and PB at the same time of lymph node pathology. We found that the lymphocytes in the LN highly expressed CD20 (Figure 2) and the expression of CD2, CD3, CD5, CD7, HLA-DR, CD10, CD34, CD8, CD23, FMC7, CD79b, CD79a, and CyCD3 did not show any difference (data not shown).

**Figure 3:**
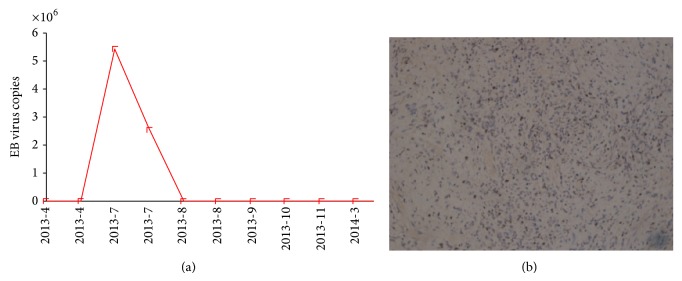
Epstein-Barr virus infection. (a) Detection of the Epstein-Barr virus by PCR. EBV copies were shown from 2013.4 to 2014.3. (b) The biopsy of EBV.

**Figure 4:**
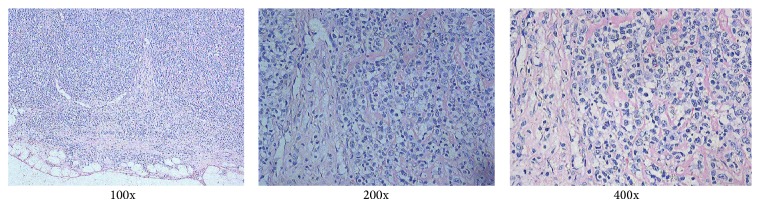
Pathology of PTLD. The pathology slides were stain of H&E by standard procedure. Pathology of PTLD was shown separately (100x, 200x and 400x).

On day 12, the serum of the patient showed high DNA copies of EB virus (Figure 3(a)), which was consistent with the LN biopsy later on (Figure 3(b)). As the pathology still took time to develop, this patient received preemptive rituximab therapy by high suspicion of PTLD. We initiated the preemptive therapy with a course of 1 dose of rituximab (375 mg/m^2^) as well as reducing and withdrawing immunosuppressant reagent. After PTLD was diagnosed by pathology, the other 3 doses of rituximab (375 mg/m^2^, qw) were given accordingly. After rituximab treatment, the patient showed absence of fever, reduction in lymph node size, and no detectable DNA copies of EB virus (Figure 3). One week after LN biopsy, the diagnosis of PTLD was confirmed by pathology, which was different from the primary disease AITL (Figure 4). The PTLD was monomorphic, and the morphology is consistent with diffuse large B cell lymphoma. Immunohistochemistry showed granzyme B, CXCL13, and PD-1 positive; TIA-I and perforin scattered positive; bcl-6 negative. Ki-67 was about 90% positive. These results indicated that Lymph node flow cytometry could be a prompt technique in recognition of PTLD.

Twenty months after HSCT, with a mild limited chronic graft-versus-host disease (I-II), the patient still maintains a complete remission (CR) well without any symptoms of AITL or PTLD.

## 3. Discussion

PTLD is one of the lethal complications of HSCT. The median onset of disease in HSCT recipients is 70–90 days. However, it has been reported that PLTD can happen as early as 1 week and as late as 9 years post transplantation [12]. Here, we report a successful treatment of ultra early developed PTLD after HLA-matched sibling HSCT with rituximab by the application of lymph node flow cytometry as a prompt recognition of the disease.

The key of early diagnosis of PTLD is active awareness. In general, any patient showing adenopathy, mass lesions, fever, unexplained pain, or weight loss after HSCT should be estimated of PTLD. Due to the atypical signs and symptoms of PLTD, the differential diagnosis should include infection, lymphoma, and engraftment syndrome, etc. To clarify the diagnoses, tissue biopsy confirmation of PTLD is required.

In most cases, PTLD is a B cell disease, which in pathology can have various manifestations. In general, PTLD should refer to the neoplastic end of the PTLD spectrum. Majority of the B cell neoplasm is believed to be the result of EBV infection [13]. Also, in clinic, most PTLDs after stem cell transplantation are EBV positive. The EBV reactivation and EBV lymphoproliferative diseases have been increasingly observed in recipients who received ATG for the prophylaxis of GVHD, or a reduced intensity conditioning regimen. We monitored the EBV viral load in peripheral blood. And we found that the EBV copies are associated with PTLD status in this case.

PTLD after HSCT is predominantly derived from donor B cells and typically occurs within 6 months after transplantation, before reconstitution of the EBV-specific cytotoxic T lymphocyte (CTL) response. However, recent reports suggested that in pediatric patients who received reduced intensity conditioning regimens that included ATG or Campath, PTLD may be a consequence of persisting recipient-derived B cells. It will be interesting to know whether PTLD was derived from donor cells or recipient cells in our case if we could perform staining and confirm from confocal Laser scanning microscope.

The treatment of PTLD includes antiviral therapy, reduction of immunosuppression, local treatment (surgery), cytokine therapy, cytotoxic chemotherapy, cellular immunotherapy, and anti-B cell antibodies. The mortality of PTLD remains high. The key to better treatment response relies on early diagnosis and early involvement. Still, reduction in immunosuppression remains the primary therapy for PTLD. In the present case, we reduced immunosuppression drugs the moment we suspected PTLD and soon discontinued them.

CD20 is expressed on the surface of all B-cells beginning at the pro-B phase and progressively increasing in concentration until maturity. Its function is to enable optimal B-cell immune response, specifically against T-independent antigens [14]. CD20 is the target of rituximab. There are reports showing that rituximab is good for controlling B-cell proliferation [9, 14, 15]. It is promising that some patients achieving long-term relapse-free survival. In this case, we applied high expression of CD20 in the LN as the indication of rituximab for the patient. And the therapeutic effect of this anti-CD20 treatment is oblivious, suggesting that rituximab is a good choice for the patient with PLTD. Also, statistics show that multivisceral disease, late-onset PTLD and CNS involvement might be poor predictors to rituximab. In the long term follow-up of the present case, we found that the patient display a long-term shortage of serum gamma globulin, which might result from the usage of rituximab and/or ATG.

In conclusion, we report an ultra early developed PTLD case after HLA-matched sibling HSCT. This case is a successful treatment by using rituximab. Rapid test by using flow analysis can overcome the shortage of late report of LN pathology biopsy and contribute to early and successful treatment of PTLD. Therefore, early recognition of PTLD, early LN biopsy, and early diagnosis are the key to successful treatment of PTLD. Till now, there is no convincing data for the prophylaxis of PLTD, The present case suggest that for the high risk patient of PTLD, especially for those serum EBV positive patient, using rituximab early after HSCT could be a good way to prevent PTLD.
